# The “Can Do, Do Do” Framework Applied to Assess the Association between Physical Capacity, Physical Activity and Prospective Falls, Subsequent Fractures, and Mortality in Patients Visiting the Fracture Liaison Service

**DOI:** 10.3390/jpm14040337

**Published:** 2024-03-23

**Authors:** Merle R. Schene, Caroline E. Wyers, Johanna H. M. Driessen, Lisanne Vranken, Kenneth Meijer, Joop P. van den Bergh, Hanna C. Willems

**Affiliations:** 1 NUTRIM School of Nutrition and Translational Research in Metabolism, Maastricht University, P.O. Box 616, 6200 MD Maastricht, The Netherlands; 2 Department of Internal Medicine, VieCuri Medical Center, P.O. Box 1926, 5900 BX Venlo, The Netherlands; 3 Internal Medicine and Geriatrics, Amsterdam UMC, University of Amsterdam, Meibergdreef 9, 1105 AZ Amsterdam, The Netherlands; 4 Department of Internal Medicine, Maastricht University Medical Center+, P.O. Box 616, 6200 MD Maastricht, The Netherlands; 5 Department of Clinical Pharmacy and Toxicology, Maastricht University Medical Centre+, P.O. Box 5800, 6202 AZ Maastricht, The Netherlands; 6 Department of Clinical Pharmacy, CARIM School for Cardiovascular Research Institute Maastricht, Maastricht University, P.O. Box 616, 6200 MD Maastricht, The Netherlands; 7 Department of Nutrition and Movement Sciences, Maastricht University, P.O. Box 616, 6200 MD Maastricht, The Netherlands; 8 Amsterdam Bone Center, Amsterdam Movement Sciences, Amsterdam UMC, Location VUmc, De Boelelaan 1117, 1081 HV Amsterdam, The Netherlands

**Keywords:** Fracture Liaison Service, physical activity, physical capacity, falls, accelerometer

## Abstract

The “can do, do do” framework combines measures of poor and normal physical capacity (PC, measured by a 6 min walking test, can do/can’t do) and physical activity (PA, measured by accelerometer, do do/don’t do) into four domains and is able to categorize patient subgroups with distinct clinical characteristics, including fall and fracture risk factors. This study aims to explore the association between domain categorization and prospective fall, fracture, and mortality outcomes. This 6-year prospective study included patients visiting a Fracture Liaison Service with a recent fracture. Outcomes were first fall (at 3 years of follow-up, measured by fall diaries), first subsequent fracture, and mortality (at 6 years). Cumulative incidences of all three outcomes were calculated. The association between domain categorization and time to the three outcomes was assessed by uni- and multivariate Cox proportional hazard analysis with the “can do, do do” group as reference. The physical performance of 400 patients with a recent fracture was assessed (mean age: 64 years; 70.8% female), of whom 61.5%, 20.3%, and 4.9% sustained a first fall, sustained a subsequent fracture, or had died. Domain categorization using the “can do, do do” framework was not associated with time to first fall, subsequent fracture, or mortality in the multivariate Cox regression analysis for all groups. “Can’t do, don’t do” group: hazard ratio [HR] for first fall: 0.75 (95% confidence interval [CI]: 0.45–1.23), first fracture HR: 0.58 (95% CI: 0.24–1.41), and mortality HR: 1.19 (95% CI: 0.54–6.95). Categorizing patients into a two-dimensional framework seems inadequate to study complex, multifactorial outcomes. A personalized approach based on known fall and fracture risk factors might be preferable.

## 1. Introduction

The global burden of disease of fractures is high. About 50% of females and 20% of males are estimated to sustain a fracture after their 50th life year [1]. In the European Union, the number of fragility fractures alone (e.g., fractures due to osteoporosis) was estimated to be 4.3 million in 2019, resulting in a total cost of EUR 56.9 billion, and it is expected to rise in the future [2]. Fractures are associated with short- and long-term disability and a decrease in quality of life [2]. Fracture patients have a high imminent risk of falls, subsequent fractures, and mortality [3]. Therefore, adequate and early secondary fall and fracture prevention is essential to lower the risk of subsequent falls, fractures, and mortality post-fracture [4,5]. The Fracture Liaison Service (FLS) is the recommended organizational approach for fracture prevention in terms of (cost-)effectiveness [6,7,8]. At the FLS, personal risk factors are evaluated, including causes of osteoporosis, comorbidities, and medication use. FLS care ideally includes a fall assessment [4].

In FLS patients, approximately 80% of subsequent fractures are fall-related [9]. The majority of falls in older persons occur during mobility tasks, such as walking, and most falls are caused by slips and trips [10,11]. An evaluation of physical performance including mobility tasks is therefore an essential part of fall risk evaluation [12]. The “can do, do do” framework is a recent approach for an integrated assessment of physical performance. It combines two distinct, yet related, domains of physical performance: physical capacity (PC; can do, can’t do) and physical activity (PA; do, do, don’t do) into four quadrant groups [13,14]. PC is objectively measured physical performance; in the “can do, do do” framework, the 6 min walking test (6MWT) is used to test walking ability [13,15]. Impaired walking ability (e.g., abnormalities in gait or balance) has been associated with future falls [12,16,17,18,19] and fractures and post-fracture mortality (in males) [20,21]. PA is defined as “any bodily movement produced by skeletal muscles that results in energy expenditure”, and is measured by an accelerometer [13,22]. PA might protect or expose (due to higher exposure to risk) persons to falls [23,24,25,26]. In fact, some studies found a U-shaped association with both inactive and highly active adults at increased fall risk [26]. The “can do, do do” framework has proven to be a useful and practical tool to identify patient subgroups with distinct clinical characteristics [13,15,27,28]. In patients with COPD, a preserved PC was found to be associated with a lower 6-year mortality risk compared to decreased PC, regardless of PA level [29]. A recent study in patients with a fracture showed that the framework was able to categorize patient groups in terms of fall and fracture risk factors, such as fall history, fear of falling, type of fracture, and osteoporosis [15].

Thus, for fracture patients the “can do, do do” framework could possibly be of value to test physical performance in the context of future falls in order to guide personalized exercise interventions. However, the prospective application of the “can do, do do” framework to assess associated clinical outcomes is scarce. The aim of our study was to explore the association between domain categorization and future falls, subsequent fractures, and all-cause mortality in patients visiting an FLS.

## 2. Materials and Methods

### 2.1. Study Population

This study used data from the FX MoVie study, a 3-year prospective cohort study of patients visiting the FLS of VieCuri Medical Center in the south of the Netherlands. The cohort was established as follows: 1. All patients who visited the emergency room with a recent clinical fracture were invited to the FLS by a nurse specialized in osteoporosis, according to the Dutch guidelines for osteoporosis and fracture prevention [30]. Patients with a radiologically confirmed fracture, aged 50–90 years old, and living in the referral area of the hospital were invited. Patients with fractures due to high-energy trauma, osteomyelitis, or bone metastasis and patients concurrently treated for a malignancy were not invited. 2. All patients attending the FLS for fracture risk evaluation between July 2014 and June 2016 were asked to participate in the FXMoVie study. Patients with cognitive impairment, patients with peri-prosthetic fractures, and non-Caucasians were excluded from participation in the prospective study cohort (FXMoVie). Prior to participation, patients were given study information through oral and written communication, and all participants gave written informed consent. The study protocols were approved by a medical ethical committee (NL45707.072.13 and 2022-3319). For this study, patients with missing scores in the PC or PA data were excluded from the analyses.

### 2.2. Data Collection

During the FLS visit, sex and demographics were collected for all patients. Cause of the fracture and risk factors for fractures and falls were evaluated by a detailed questionnaire according to the Dutch guidelines for osteoporosis and fracture prevention [30]. This included self-reported measures on smoking and alcohol use, fall history, dizziness and balance problems, fear of falling, and use of walking aids. Information on comorbidities was derived from the electronic patient files and labeled according to ICD-10 (international classification of disease) standards [31]. Index fracture location was grouped into: I. hip fractures; II. major fractures: vertebra, multiple rib, humerus, pelvis, distal femur, and proximal tibia; and III. minor fractures: all other fractures (including finger and toe fractures), according to Center et al. [32]. Measurements included height, weight, body mass index (BMI), and bone mineral density (BMD) measurement at the lumbar spine, total hip, and femoral neck, and vertebral fracture assessment was performed using dual-energy X-ray absorptiometry (DEXA). Anti-osteoporotic treatment (AOM; e.g., bisphosphonates, denosumab, or teriparatide) was started according to the Dutch guidelines for osteoporosis and fracture prevention—in summary: in case of T score ≤ −2.5, or in case of osteopenia with at least one Grade 2 or 3 prevalent vertebral fracture according to Genant et al. [33].

### 2.3. Can Do, Do Do Framework

The “can do, do do” framework and our methods of testing PC and PA have been previously described elsewhere in detail [15]. In short, the framework combines a measure of PC with a measure of PA to form four quadrant groups: 1. preserved PC, preserved PA (can do, do do); 2. low PC, preserved PA (can’t do, do do); 3. preserved PC, low PA (can do, don’t do); 4. low PC, low PA (can’t do, don’t do). PC was measured by a trained nurse at the baseline study visit with the 6MWT. The 6MWT assesses walking ability and overall physical performance and has excellent inter-rater and test–retest reliability [34,35]. Patients were asked to walk as far as possible, up and down a 10 m walkway, rest when needed, and the use of walking aids was permitted. No encouragements were given during the test. Sex-stratified cutoff scores for poor or normal performance were based on Beekman et al. and amounted to a cutoff at 385 m for males and 366 m for females [36]. PA was measured by the MOX activity monitor (Maastricht Instruments B.V., The Netherlands). The MOX is a small, waterproof device that was attached to the right thigh. PA was measured during eight consecutive days in which patients were asked to follow normal daily activity routines. Details on the properties, processing of data, activity classification, calibration, and validity are described in detail elsewhere [15,37,38]. Static and dynamic activity (DA) were determined using the signal magnitude area: a measure of the intensity of physical activity. DA (average minutes per day) was further categorized as low physical activity (LPA, <3 metabolic equivalents [METS]), moderate-to-vigorous physical activity (MVPA, 3–6 METS), and vigorous physical activity (VPA, >6 METS) [38], and the threshold for poor or normal PA was defined according to WHO recommendations on minimum weekly exercise for general health benefits at <150 min of MVPA/VPA per week [39].

### 2.4. Outcome Measures

The outcomes were time to fall, time to first subsequent fracture, and time to death (by any cause). Registration of falls, subsequent fractures, and mortality started after inclusion in the study (baseline), a mean (SD) 3.5 (0.99) months after the index fracture. Initial prospective follow-up of participants was three years. A fall was defined according to the WHO definition as “an event which results in a person coming to rest inadvertently on the ground or floor or other lower level” [40]. Falls were recorded by participants in weekly fall diaries, which were returned by postal service. Research assistants verified and checked the diaries for completeness at three and six months by telephone and during the regular study visits at one, two, and three years of follow-up. Subsequent fracture incidence and mortality were evaluated at the yearly study visits at one, two, and three years of follow-up. All subsequent fractures were radiologically confirmed by radiology reports. All reported vertebral fractures were clinical vertebral fractures, as throughout follow-up no routine imaging of the spine was performed. The follow-up period for the outcomes of subsequent fractures and mortality was extended until 6 years after index fracture for all patients. Fall data were not available for the period between 3 and 6 years of follow-up.

### 2.5. Statistical Analysis

Descriptive analyses were performed for the whole population and predefined quadrant groups separately. Continuous data are presented as mean and SD for normally distributed data and median and IQR for non-normally distributed data. Categorical data are presented as frequencies and percentages. Cumulative incidences of first fractures, first falls, and mortality were assessed at 1, 2, 3 (fractures, mortality, falls), and 6 years (only fractures and mortality) of follow-up for the total population and the different “can do, do do” quadrants. Outcomes were also stratified by sex and compared using chi-square or Fisher exact tests in cases of low expected counts. Cox proportional hazard regression models were used to calculate crude and adjusted hazard ratios and 95% CIs for first subsequent fractures, first falls, and mortality for the different “can do, do do” quadrants. The “can do, do do” group was used as the main reference category, but all other between-group comparisons were also carried out. Patients were followed from baseline to censoring: outcome of interest (i.e., first fall, first fracture, or death), or end of data collection, whichever came first. For the outcome “fall” and “fracture”, patients were censored in case of death during follow-up. For the outcome “fall”, censoring was also applied if the patient was lost to follow-up (defined as the moment when >1 week of fall data were missing). Multivariate models were adjusted for a predefined set of variables based on the literature, including age, sex (all outcomes), BMI, lowest BMD, and vertebral fractures (only for fracture outcome). Proportional hazards assumption was checked by visual assessment of LML plots and by performing time program functions with all quadrants and all covariables independently. Additional analyses included Cox proportional hazards regression in which PC and PA were entered as continuous variables, as well as their interaction term. As a sensitivity analysis, a competing risk analysis was performed using the cumulative incidence competing risk method, according to Fine and Grey [41], in which death was considered a competing risk for the outcome of first fall or fracture. However, only one death occurred during the first 3 years of follow-up, which was censored due to incomplete fall data of the patient. Composite endpoints at 3 years (all outcomes) and 6 years (fractures and mortality) were explored visually and compared between the poor and normal PC and PA groups and for the patients in the 1st quartile of PC and PA compared to the highest three quartiles. A *p*-value of <0.05 was considered statistically significant. The competing risk analysis for the outcome of first fracture was carried out in R statistical software (version 4.2.1, R Core Team, 2022, R Foundation for Statistical Computing, Vienna, Austria), and R studio (RStudio Team, 2020, RStudio: Integrated Development for R. RStudio, PBC, Boston, MA) using the “cprsk” package. All other statistical analyses were performed in SPSS (version 28, 2021, IBM SPSS Statistics for Windows, Armonk, NY: IBM Corp.)

## 3. Results

### 3.1. Baseline Characteristics

Baseline characteristics are presented in Table 1. Participants were unequally divided into groups, with the largest proportion of 69.5% of the participants included in the “can do, do do” group, and smaller proportions in the other groups: 19.3% in the “can’t do, do do”, 3.0% in the “can do, don’t do”, and 8.3% in the “can’t do don’t do” group (*p* < 0.001). Participants in the “can do, do do” group were younger, had less severe fracture types, had less often osteoporosis or a prevalent vertebral fracture and had a treatment indication for AOM less often, had fewer comorbidities and fewer fall risk factors including fear of falling, and higher scores on the physical performance tests.

A total of five male patients and seven female patients had >1% missing fall data and were excluded from the cumulative incidence analyses. Table 2 shows the cumulative incidence of first fractures, first falls, and mortality during follow-up, stratified by year of follow-up and sex.

### 3.2. Time to First Fall

In total, 61.5% of the participants with sufficient fall data (N = 388) experienced at least one fall during follow-up of 3 years (Table 2). No significant association was found between quadrant groups and time to first fall during the 3 years of follow-up in the uni- and multivariate analysis (Table 3). Between-group comparison using alternative reference groups also did not show significant associations. A sensitivity analysis using dichotomous outcomes—“can do” vs. “can’t do” and “do do” vs. “don’t do”—did not show significant associations of low PC or low PA with time to first fall.

### 3.3. Time to First Subsequent Fracture

In total, 20.3% of the participants had sustained a first subsequent fracture at 6-year follow-up (Table 2). No significant association was found between each of the quadrant groups and time to first subsequent fracture during the 6 years of follow-up (Table 3), nor in the between-group analyses. The sensitivity analyses with death considered as a competing risk did not show a significant association. Sensitivity analysis using the dichotomous outcomes “can do” vs. “can’t do” and “do do” vs. “don’t do” and continuous outcomes of PC and PA measures did not show a significant association with time to first fracture.

### 3.4. Time to Death

During 6 years of follow-up, 4.8% of the participants had died. The proportion of participants that died was significantly different between quadrant groups at six years of follow-up: “can do, do do”: 2.9%, “can’t do, do do”: 9.1%, “can do, don’t do”: 0%, and “can’t do, don’t do”: 12.1% (*p* = 0.019, Table 2). Compared to the reference group, the “can’t do, do do” quadrant group was also significantly associated with time to death during 6 years of follow-up in the univariate analysis for the “can’t do, do do” group: HR = 3.29 (95% CI 1.19–9.07) and the “can’t do, don’t do” group: HR = 4.40 (95% CI 1.33–14.62). However, this association did not remain significant in the multivariate analysis (Table 3), nor did any of the between-group analyses yield significant results. A sensitivity analysis using the dichotomous outcomes “can do” vs. “can’t do” and “do do” vs. “don’t do” showed a significant association between the “can’t do” group: HR = 3.73 (95%CI 1.50–9.28) with time to first fracture compared to the “can do” group in univariate analysis, but this association did not remain significant in the multivariate analysis adjusted for age and sex: HR = 1.75, (95% CI 0.65–4.70).

### 3.5. Composite Outcomes

**Figure 1:**
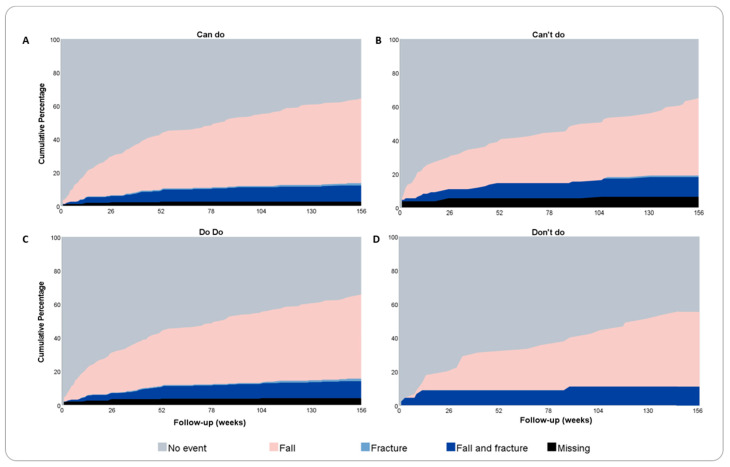
Composite endpoints during three years of follow-up. (**A**–**D**) Visualization of composite endpoints falls and fractures during 3 years of follow-up (in weeks) for normal and poor physical capacity and activity groups. The cutoff for low physical capacity is 366 m for females and 385 for males (can’t do); the cutoff for low physical activity is an average of 21 min/day (don’t do).

**Figure 2:**
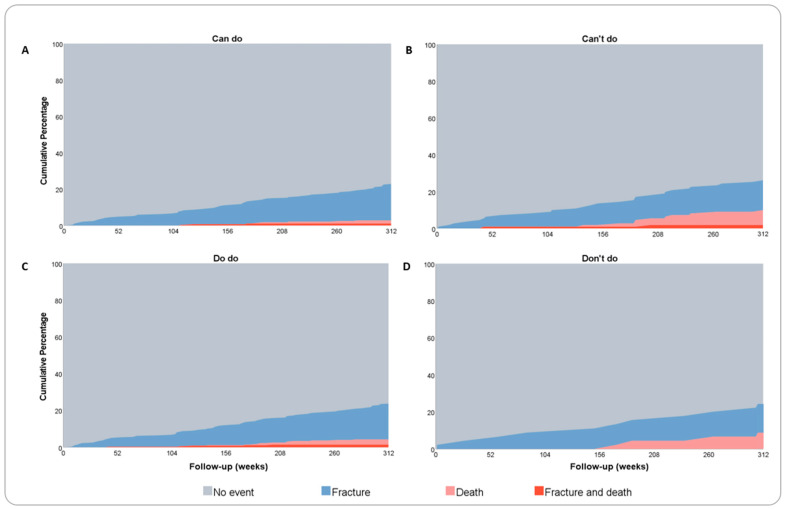
Composite endpoints during six years of follow-up. (**A**–**D**) Visualization of composite endpoints fractures and mortality during 6 years of follow-up (in weeks) for normal and poor physical capacity and activity groups. The cutoff for low physical capacity is 366 m for females and 385 for males (can’t do), the cutoff for low physical activity is an average of 21 min/day (don’t do).

The visualization of composite endpoints are presented in Figure 1A–D and Figure 2A–D. Figure 1A–D displays the outcome of the composite endpoints falls, fractures, and mortality at 3 years of follow-up for the “can do” and the “can’t do” groups and the “do do “and “don’t do” groups, respectively. Figure 2A–D displays the outcome of the composite endpoints of fractures and mortality at 6 years of follow-up for the “can do” and the “can’t do” groups and the “do do “and “don’t do” groups, respectively. For both endpoints, the composite outcomes did not differ between groups. Quadrant group categorization based on quartile distribution of PC and PA measures instead of categorization based on predefined thresholds did not show differences in composite endpoint visualization.

## 4. Discussion

In this prospective study of 400 patients visiting a FLS with a recent fracture, we did not find a significant association between physical performance at 3, 5 months after fracture, assessed with the “can do, do do” framework, and time to first fall, first fracture, or death.

A previous cross-sectional assessment of the “can do, do do” framework showed differences in fall and fracture risk factors between the quadrant groups; patients in the can’t do, don’t do” group were older; presented with more major index fractures; more often had osteoporosis, prevalent vertebral fractures, and comorbidities; scored lower on all physical performance tests; and more often used a walking aid [15]. Contrarily, our study did not show an association with the clinically relevant outcomes of first fall (at three years), first fracture, and mortality (at six years). Our results are also in contrast with studies in the general older population associating poor physical performance (including both PC and PA measures) with future falls [12,25], subsequent fractures [42,43,44], and mortality [39,45,46,47,48]. Studies in fracture populations are fewer in number, but several involving older individuals with hip, radius, or any low-trauma fracture have reported associations between poor PC (using balance and gait measures) and falls [49,50,51,52] and post-fracture mortality in older males [52]. Poor PA has been associated with subsequent fractures in older males [53,54], but not females [55], after fracture. Interestingly, one study reported that males with a recent fracture who sustained a subsequent fracture had higher PA and gait speed measures compared to those who did not sustain a subsequent fracture [52]. This suggests that they may be more likely to engage in risky behavior. However, comparability between the studies in fracture patients is low, as they differ in terms of the age and sex of the included population, index fracture type, timing of the measurement before or after fracture, and measurement method of PC (different performance tests were evaluated) and PA (mostly self-reported with questionnaire) [52,54]. Only one study previously explored the “can do, do do” framework prospectively. Vaes et al. explored mortality risk in the different quadrant groups in patients with COPD [29] and found that patients with preserved PC had significantly lower 6-year mortality risk than those with poor PC (HR = 0.36, 95% CI; 0.14–0.93). In our study, PC (can do vs. can’t do) was associated with mortality in the univariate but not multivariate analysis. However, in the study by Vaes et al. the threshold values were study population driven and not based on pre-determined cutoff scores for poor or normal PC and PA. The lack of association with mortality outcomes in our study might be explained by the low mortality incidence during follow-up. The survival of 95.5% was the same as the 6-year survival of 65-year-olds in the general Dutch population (95.8%) [56], indicating a healthy subset of fracture patients, who generally have an increased mortality risk [3].

The absence of associations in our study might also be attributed to the unequal distribution of participants in the framework that resulted in a small number of participants included in the “can do, don’t do” and “can’t do, don’t do” groups. This was partly due to the low proportion of patients with low PA. The use of predefined cutoff scores for poor and normal PA (<150 min/week and 21/min per day MVPA/VPA) was based on the WHO recommendation to prevent adverse health outcomes, such as falls and fall-related injuries and all-cause and cause-specific mortality [39,57]. These are in line with other cutoff scores: <25.1 min MVPA/day was associated with a higher fall risk in older (70+) females [58]; and in community-dwelling older males, those with a history of >2 falls had an average of 24 min MVPA/day compared to 40 min/day in non-fallers [59]. In our study, the assessment of composite endpoints using the quartile distribution (instead of predefined cutoff scores) did not show superior discrimination between groups. Thus, the chosen cutoff scores do not seem to have had a great influence on the absence of an association.

More likely, our study population is too homogeneous, as it consists primarily of individuals who have sustained a fracture and are nearly all “previous fallers”. In 86.5% of our study population, the fracture that led to FLS evaluation was caused by a fall, and this did not vary between quadrant groups. Patients with a recent fracture are at a high risk for subsequent falls and fractures, with 38% of our participants experiencing a fall in the first year and 60% in the first three years, and 21% sustaining a subsequent fracture during six years of follow-up, despite evaluation and treatment according to best practice standards. These incidences did not differ between quadrant groups. Therefore, our study suggests that measures of PC and PA, measured during the FLS visit, do not differentiate enough between patients with a recent fracture to show any association with the clinical outcomes of falls, fractures, and mortality—a conclusion that was also suggested in a previous study assessing physical performance and subsequent fracture risk [52].

To effectively evaluate the likelihood of future fractures, it is crucial to assess fall risk factors, as falls are a significant predictor. A comprehensive review of the World Falls Guidelines emphasized the multifactorial nature of fall prediction and concluded that no single measurement can guarantee a high level of confidence in fall risk assessment for older adults. The review recommended both balance and gait assessments in fall prediction [12,17]. They further underlined that mobility assessment is only one part of fall prediction, and many other factors (e.g., fall-risk-increasing drugs, cardiovascular management, and cognitive function and sensory function decline) are also important [12]. Consequently, fall prediction and prevention is highly personalized. Interventions, including physical performance interventions, need to target multiple domains and need to be tailored to the patients’ needs [12,60,61]. Our study underlines this; even though the “can do, don’t do” framework offers a more complex approach compared to single physical function tests, this framework remains on one hand too “simplistic” to capture the multifactorial causes of falls and fractures. On the other hand, in testing and constructing combined measures, we lose information for a tailor-made, individual evaluation of different physical performance domains and corresponding treatment options. Thus, the categorization into a two-dimensional framework is inadequate to study complex clinical outcomes such as falls, fractures, and death, and a personalized approach based on existing fall and fracture risk factors from international guidelines [4,12] seems preferable.

### Strengths and Limitations

One of the key strengths of our study is the inclusion of a sizable cohort of patients who had recently experienced a fracture, along with the examination of three clinically relevant outcomes. Secondly, this study is one of the first to assess physical performance in fracture patients in terms of clinical outcomes. Another strength is that we used a competing risk analysis to check the robustness of our results. Failure to adjust for the competing risk of death would result in an overestimation of the risk of outcome [41]. However, several limitations apply to this study. First, this was an explorative study and results will have to be confirmed in other prospective studies. Second, as stated, our cohort is relatively healthy compared to the wider range of fracture patients presenting at the Emergency Room, which might be reflected in the low number of patients with poor PA. The fracture patients that visit the FLS are only 60% of all consecutive patients with a clinical fracture [15], and previous studies showed that those who visit the FLS are more often female, younger, less frail, and less likely to have a hip fracture compared to those who do not attend [62,63]. Moreover, the participation in this prospective study was voluntary, and mostly younger patients with less severe types of fracture were willing and able to participate. The low incidence of mortality results in a lower reliability for this outcome, limited options of covariables in the multivariable models, and limited added benefit of the competing risk analysis. Lastly, we did not account for changes in physical performance parameters and patients’ exercise and rehabilitation activities during follow-up. Some studies suggest that the patients’ change in physical performance over time is equally, if not more, important to predict falls, fractures, and mortality as single test values [42,52]. Future studies should assess the association between physical performance measures and fall, subsequent fracture, and mortality risk in larger cohorts of fracture patients, and assess changes in performance over time.

## 5. Conclusions

The “can do, do do” framework was not associated with time to fracture, time to fall, or time to death in a 6-year prospective study. This study suggests that categorizing patients into a two-dimensional framework is inadequate to study complex, multifactorial outcomes and that a personalized approach based on individual assessment of existing fall and fracture risk factors might be preferable.

## Figures and Tables

**Table 1:** Baseline characteristics.

	Total Population	Can Do, Do Do	Can’t Do, Do Do	Can Do, Don’t Do	Can’t Do, Don’t Do	*p*-Value
Number of patients ^b^	400 (100)	278 (69.5)	77 (19.3)	12 (3.0)	33 (8.3)	<0.001 *
Females ^b^	283 (70.8)	191 (67.5)	53 (18.7)	12 (4.2)	27 (9.5)	<0.001 *
Males ^b^	117 (29.2)	87 (74.4)	24 (20.5)	0 (0)	6 (5.1)	<0.001 *
Age ^a^	64.6 ± 8.6	62.7 ± 7.9	68.6 ± 9.0	67.4 ± 8	70.4 ± 8.2	<0.001 *
BMI ^a^	27.2 ± 4.4	27.3 ± 4.2	29.0 ± 4.2	26.2 ± 5.0	27.2 ± 6.0	0.02 *
Time since fracture (days) ^a^	108.0 ± 30.2	107.1 ± 29.2	110.6 ± 31.4	105.7 ± 30.3	110.4 ± 35.8	0.77
Alcohol use ^b^	316 (79.0)	239 (86.0)	50 (64.9)	7 (58.3)	20 (60.6)	<0.001 *
Smoking ^b^	269 (67.3)	190 (68.3)	54 (70.1)	4 (33.3)	21 (63.6)	0.08
Fracture type ^b^						<0.001 *
Hip	20 (5)	6 (2.2)	5 (6.5)	1 (8.3)	8 (24.2)	
Major	80 (20)	39 (14.0)	22 (28.6)	4 (33.3)	15 (45.5)	
Minor	250 (62.5)	191 (68.7)	45 (58.4)	6 (50.0)	8 (24.2)	
Finger and toe	50 (12.5)	42 (15.1)	5 (6.5)	1 (8.3)	2 (6.1)	
BMD ^b^						<0.001 *
Normal BMD	112 (28.0)	87 (31.3)	21 (27.3)	2 (16.7)	2 (6.1)	
Osteopenia	200 (50.0)	142 (51.1)	40 (51.9)	5 (41.7)	13 (39.4)	
Osteoporosis	88 (22)	49 (17.6)	16 (20.8)	5 (41.7)	18 (54.4)	
≥1 Prevalent VF gr 2–3 ^b^	49 (12.3)	23 (8.3)	15 (19.5)	1 (8.3)	10 (30.3)	<0.001 *
AOM treatment ^b^	135 (33.7)	79 (28.4)	24 (31.4)	7 (58.3)	25 (75.8)	<0.001 *
Cardiovascular comorbidity ^b^	163 (40.8)	99 (35.6)	41 (53.2)	2 (16.7)	21 (63.6)	<0.001 *
Asthma/COPD ^b^	40 (10.0)	21 (7.6)	12 (15.6)	0	7 (21.2)	0.019 *
Diabetes ^b^	29 (72)	13 (4.7)	10 (13.0)	0	6 (18.2)	0.006 *
Fracture caused by a fall ^b^	346 (86.5)	241 (86.7)	66 (85.7)	11 (91.7)	28 (84.8)	0.97
≥2 Falls in past year ^b^	51 (12.8)	32 (11.6)	14 (18.2)	1 (8.3)	4 (12.1)	0.46
Dizziness–balance ^b^	94 (25.3)	52 (20.2)	25 (34.2)	5 (45.5)	12 (40.0)	0.006 *
Walking aid ^b^	15 (3.8)	2 (0.7)	7 (9.1)	0	6 (18.2)	<0.001 *
Fear of falling ^b^	39 (9.8)	16 (5.8)	15 (19.7)	0	8 (24.2)	<0.001 *
MVPA-VPA (Av. min/day) ^a^	56 (26.3–87.0)	70 (48–100)	43 (32–59)	12 (11–18)	14 (8–18)	<0.001 *
6MWD (m) ^a^	428 (364.3–489.8)	465 (420–510)	330 (299–354)	419 (396–476)	280 (218–330)	<0.001 *

^a^ Continuous variable: mean ± SD, median (IQR); ^b^ categorical variable: number (%). AOM: anti-osteoporotic medication, BMI: body mass index, BMD: bone mineral density, 6MWD: six-minute walking distance, MVPA: moderate-to-vigorous physical activity, VF: vertebral fracture, VPA: vigorous physical activity, Av.: average. The cutoff for low physical capacity is 366 m for females and 385 for males (can’t do), the cutoff for low physical activity is an average 21 min/day (don’t do). * statistically significant difference between groups.

**Table 2:** Cumulative incidence of first fractures, first falls, and mortality.

	Can Do, Do Do	Can’t Do, Do Do	Can Do, Don’t Do	Can’t Do, Don’t Do	*p*-Value
Number of patients	278 (69.5)	77 (19.3)	12 (3.0)	33 (8.3)	
First fall by follow-up †					
1 year	112 (40.3)	28 (40.0)	5 (41.5)	8 (24.2)	0.31
2 years	146 (53.5)	35 (50.0)	6 (50.0)	13 (39.4)	0.49
3 years	170 (62.3)	45 (64.3)	7 (58.3)	18 (54.5)	0.78
First fracture by follow-up					
1 year	14 (5.0)	5 (6.5)	0 (0)	2 (6.1)	0.84
2 years	19 (6.8)	5 (6.5)	0 (0)	4 (12.1)	0.80
3 years	32 (11.5)	9 (11.7)	0 (0)	5 (15.2	0.65
6 years	60 (21.6)	14 (18.2)	1 (8.3)	6 (18.2)	0.76
Mortality by follow-up					
1 year	0 (0)	0 (0)	0 (0)	0 (0)	-
2 years	0 (0)	1 (1.3)	0 (0)	0 (0)	0.30
3 years	0 (0)	1 (1.3)	0 (0)	0 (0)	0.30
6 years	8 (2.9)	7 (9.1)	0 (0)	4 (12.1)	0.019 *

All cumulative incidences are presented as numbers (%). The cutoff for low physical capacity is 366 m for females and 385 for males (can’t do), the cutoff for low physical activity is an average of 21 min/day (don’t do). † Twelve participants were excluded due to >1% missing fall data. * Statistically significant at *p* < 0.05.

**Table 3:** Association of “can do, do do” groups with falls, subsequent fractures, and mortality.

	Time to First Fall		Time to First Subsequent		Time to Death	
			Fracture			
	Univariate	Multivariate	Univariate	Multivariate	Univariate	Multivariate
	HR 95% CI	HR 95% CI	HR 95% CI	HR 95% CI	HR 95% CI	HR 95% CI
Can do, do do (ref)	-	-	-	-	-	-
Can’t do, do do	0.99 (0.71, 1.38)	0.99 (0.70, 1.39)	0.86 (0.48, 1.53)	0.80 (0.43, 1.48)	3.29 (1.19, 9.07) *	1.55 (0.53. 4.56)
Can do, don’t do	0.74 (0.41, 1.88)	0.83 (0.39, 1.78)	0.34 (0.05, 2.48)	0.25 (0.04, 1.84)	-	-
Can’t do, don’t do	0.76 (0.47, 1.24)	0.75 (0.45, 1.23)	0.88 (0.37, 2.04)	0.58 (0.24, 1.41)	4.40 (1.33, 4.62) *	1.19 (0.54, 6.95)

Time to first fall was assessed during 3 years of follow-up; time to first subsequent fracture and death was assessed during 6 years of follow-up. HR: hazard ratio. 95% CI: 95% confidence interval. Multivariate models included the following covariates: First falls: age and sex. First subsequent fractures: age, sex, BMI, prevalent VFs, and lowest BMD. Mortality: age and sex. * Statistically significant compared to reference group “can do, do do” at *p* < 0.05. Between-group comparisons with different reference groups did not yield significant results.

## Data Availability

Research data are not shared due to privacy or ethical restrictions.
